# What Can We Do to Be Saved?

**Published:** 1913-02

**Authors:** Samuel A. Visanska

**Affiliations:** Atlanta, Ga.


### "WHAT CAN WE DO TO BE SAVED?"

By Samuel A. Visanska, Ph. G., M. D. Atlanta, Ga.

If I were a minister of the gospel appearing before you tonight with this phrase as a text from which to preach an eloquent sermon leading toward spiritual salvation, I would not expect much attention from my hearers. Of course no ministers admit this, but to us who see more of the physical than we do of the spiritual side of men, it must be plain that human nature is so constituted that personal salvation, just for ourselves individually, seems somehow an assured fact to almost every one of us. This characteristic makes it difficult for the exponent of Christianity or for the exponent of any doctrine of change and of improvement to make an immediate impression on his hearers. I am afraid that for this reason, too, it will be hard for me to induce the members of this association to feel that the entire organization is losing wonderful opportunities for advancement, not only as to its individual members, but of the profession itself.

Heretofore, what has been the object of the association, any way? I have been a member for a long time both of the local and state organization and I cannot detect, even with careful thinking, a single thing that either of them have accomplished. Of course if I am wrong in this and the members of both organizations have been working in secrecy and quiet toward some great end which is visible only to the individual vision, then I apologize in advance; but from the viewpoint of the ordinary member, I ask, "What are we doing to warrant our existence at all?"

In the first place, if we pose as merely a semi-social organization, with the object of promoting good feeling, fellowship and mutual interest in each others' work and ambition, then there would be some hope for us; but, as we must admit if we are honest with ourselves, there is absolutely no sense of brotherhood or good feeling among our members. We are guilty of the unpardonable sin in an organization, of selecting a few personal favorities and friends who fraternize together with no attempt of the general good of the profession and are really beset with petty envy and jealousies of each other. In fact, I venture to assert broadly and positively that there is more professional jealousy among physicians than among the members of any other of the learned professions. I have never been able to understand *why* this is so or why it should seem to gain strength as the physician mounts higher in his profession, yet it is an undisputable fact. The established members of the profession who could easily afford to reach down the helping hand to those younger aspirants who stand with unsteady feet on the first rung of the ladder, refuse to even see the outstretched hand or to hear even the echo of an appeal for recognition which the younger voices might long to make.

Is this the proper ethical attitude? Would it lead to the strengthening of the profession as a whole? Would it help the individual physician to more personal success if he shuts out the younger men? I think not, myself, and I wonder what you, my brother physicians, think of it?

Then, too, the colleges at which these younger men study take their attitude of indifference from the profession itself; they turn a deaf ear and a cold glance on the young graduate; they are even indifferent to the fate of these young men when they leave the college and commence their hard fight for place and prominence in the profession. It seems to me that an Alma Mater should in very truth perform some of the services of a *real* mother and at least endow her children either with some supervision or with some indorsement as to their capabilities. It would certainly be a good piece of work for this association to undertake a sort of guardianship of young graduates in the profession of medicine. We might even urge the colleges to consider their own students when positions are open in institutions or when the older doctors apply for help in the hospitals and occasionally in private practice. Under present conditions, it is considered quite proper for the medical college and the established physician to disclaim any knowledge of the ability of the young graduate, thus thrusting him into practice without the aid of an encouraging word. Surely, there is room for reform in this particular.

Again, I ask what have we done as a medical society for the good of the community in which we live? What sanitary laws have we advocated? What reforms have we instituted; where, in short, have we done a single thing to justify our existence as a body of educated medical men who should have the best interests of the community at heart? If there has ever been a single good thing emanuate from this organization I would like to have a printed history of the movement so that I might frame it and hang it in full view; I have looked over the ground carefully and I cannot find one thing which could come under this head.

You may say, as an individual, "Well, I have treated Smith, Brown or Robinson and have made no charge." Do you think that a laudable action? Well, I do not. It is just such services on the part of the physician which have gone far toward pauperizing the people. Of course where real charity is needed; when actual poverty prevents payment of doctor's bills, I am the last person to advocate harshness on the part of the physician. On the contrary, we all do a lot of charity practice and are glad to do it. We serve on free clinics, we travel miles to treat free patients, but I do advocate the distinction between those *unable* to pay and those merely *unwilling* to do so.

We treat hundreds of patients each year who belong to the latter class. It seems to me that many people regard the physician pretty much as they do the government and think it perfectly legitimate if they can get out of paying a doctor's bill. Why this should be so I do not know, unless the original fault lies with the doctors themselves, who blindly follow some foolish fetish of so-called "charity" in treating persons who are able to pay even a small fee. There is scarcely a working man or woman to-day who cannot pay something to his physician, and the services rendered those who pay for them are much more appreciated than if they had been given gratuitously. Even in the hospitals it would be a safer and saner plan to exact a small weekly payment wherever possible. As small a sum as twenty-five cents a week gives the individual a sense of independence and makes him appreciate what he is getting more than if it were offered him perfectly free. This is one point which medical associations should consider and by a certain unity in their efforts they would eventually educate the public to an appreciation of the value of medical attention and with this appreciation would naturally follow a desire to pay for such services.

I think there should be a commission appointed to work out some practical plan along this line, and such a plan would prove helpful to the "charity" patient as well as to the physician at large. I knew a certain member of our profession who was a personal friend of my own who used to boast that he never sent a bill to any one. Of course he was well beloved; when he died he was widely eulogized, and then—well, then his family were left destitute, his children were uneducated and in danger of becoming charges of the state. Did the persons who owed him thousands of dollars come forward with help for that widow and those orphans? Not a bit of it; they were let severely alone and their sad plight has been an object lesson to me ever since.

While I cannot *urge* that physicians combine together to set a scale of fixed charges, yet I do think a medical association could do much to elevate the profession if they agreed, as a body, to *insist* on the payment of bills when such payment is possible, for every time you fail to collect your bill you entail a similar obligation on some other member of the profession who may be much less able to bear it than yourself.

It is this need of protection, of unity of combined action that I would urge on the association. Laboring men have their unions and their work is respected and appreciated; why should they rank lower in public estimation than the physcian who gives years of his life to education and training and who is then expected to lavish his hard earned knowledge on an unappreciative public?

In the past, the sons of physicians were often themselves medical men and lived at home; this lessened their expenses and they disregarded bills; but all that is changed now. The cost of living has increased 50% and how do we as physicians, meet this advance? Are we fair or just to our own families in our attitude to our debtors? I think you will all agree with me in answering NO, to this question. Our former president said we worked hand in hand with the Board of Health. But do we? I am told a number of our members have to be actually threatened before they reported births, and they even disregard the grave menace to the public by their failure to report contagious diseases to the Board of Health. Not long ago the local Board of Health passed a law requiring a card placed on the door of every house in which there was a case of typhoid fever. In a few weeks some of the doctors of our society had this law repealed. What their object was I have never been able to find out, unless it was to save possible inconvenience to a few influential patients! But I am sure that such vacillating methods must weaken our hold on the public and cause us to lose respect and confidence. I shall not discuss the need for isolating typhoid fever homes; the Board of Health considered that there was such a need and the physicians should have at least abided by this decision.

There is the grave question of pure milk, for instance. Atlanta has no certified milk; Louisville has; help us to get it. Of course there are laws governing milk and while all laws are at times inconvenient, they are made for some wise purpose and should be observed. Have these laws the full indorsement and support of our association at all times? This question is one which we must answer for ourselves as individuals and I hope we may answer them honestly.

I say we must answer this question as individuals, but after all it seems to me we will have to answer a good many questions that way, for as members of this association we seldom get together as a body in numbers large enough to really accomplish anything. We have, I am told, a membership of some two hundred; did you ever see as many as a hundred present at these meetings? I think thirty is about the average number, and thirty men from a possible two hundred must necessarily feel that their influence is lessened by just about one hundred per cent. Can we find any reason for this lack of interest in our association? Is it because you may be a graduate of Johns-Hopkins and I may not be? Is it that you are on some medical board, some hospital staff, some college faculty where I do not figure? Oh! perish the though of personal jealousy or personal pride of place! Surely that could not influence sane medical men. I sincerely hope it does not—but who shall say?

Don't you think it would be a good idea and would promote fellowship and interest if we invited the staff physicians from the hospitals as well as the students from the medical colleges to attend our meetings. An effectual way of doing this would be to have posted on the hospital and college bulletins titles of papers to be read at our meetings. These men are our future members, both of the local and state organization, and their interest cannot be enlisted too early in their careers. While this would insure our membership in the future, there is much which we can do to increase it in the present. I would suggest the following plan as one means toward this end:

I learn that there are three hundred regular physicians now practicing in our city and yet our membership numbers but two hundred. Isn't there some way to get the other hundred to join us? Could we not give a special banquet for this purpose, send out printed invitations to all the physicians, inclose an invitation to join our society to those not already on our list, then have speeches by our most distinguished members and thus create sufficient interest in our association in order to increase our membership.

What I would strongly urge now, however, is brotherhood, unity, amity and mutual tolerance. STOP KNOCKING; remember that every knock is often a boost for the other fellow. If we spent as much time reading on our materia medica as we do knocking our competitors, we would be better posted on the things which count most. This would mean the salvation of our work, salvation of ourselves as scientists, of our professon as a science, of our patients as individuals and of our influence as a potent force in the community in which we live. In the past the doctor ranked with the minister; one was sought to aid sick souls, the other to reconstruct sick bodies. The body is the temple of the soul—why have we so long disassociated the one from the other? Could not we, two hundred strong, band ourselves together for a new era in medical history? We live in a progressive southern city; we are at the gateway, as it were, to all the south, why should we not stand at the open door with hearts and minds open to higher and better things? Why should we not elevate the whole profession? We know that there are sins of omission as well as those of commission. I think we are guilty of both sorts. I do not exempt myself in this charge; I am as guilty as the rest of us, but at least I see where the reforms should be made. Will you help us make them? Medicine should be a profession devoted to the most beneficent ends; it should be designed to lift the race and to reconstruct the individual; it should certainly be exercised only for the "greatest good to the greatest number," and I believe that in our own salvation we will surely see the ultimate salvation of the race itself.

This lies in our hands quite as much as in the hands of the preacher and it can only be made by mutual co-operation and mutual unity of purpose. In beginning a new year in our association's work, I make this plea to each individual member. Let us work together for the general good and in so working, we must, in logical sequence, reach what is best for us as individuals as well as for us as loyal disciples of Galen and as enthusiastic followers of the gentle art of healing.

(Read before the Fulton County Medical Society, Jan. 16, 1913.)

